# Systematic Review of Commercially Available Clinical CMUT-Based Systems for Use in Medical Ultrasound Imaging: Products, Applications, and Performance

**DOI:** 10.3390/s25072245

**Published:** 2025-04-02

**Authors:** Ahmed Sewify, Maria Antico, Laith Alzubaidi, Haider A. Alwzwazy, Jacqueline Roots, Peter Pivonka, Davide Fontanarosa

**Affiliations:** 1 School of Clinical Sciences, Queensland University of Technology, Gardens Point Campus, 2 George St., Brisbane, QLD 4000, Australia; jacqueline.roots@hdr.qut.edu.au (J.R.); d3.fontanarosa@qut.edu.au (D.F.); 2 Centre for Biomedical Technologies (CBT), Queensland University of Technology, Brisbane, QLD 4000, Australia; maria.antico@csiro.au (M.A.); peter.pivonka@qut.edu.au (P.P.); 3 ARC ITTC Centre for Joint Biomechanics, Queensland University of Technology, Brisbane, QLD 4000, Australia; 4 Australian e-Health Research Centre, The Commonwealth Scientific and Industrial Research Organisation (CSIRO), 296 Herston Rd., Herston, QLD 4029, Australia; 5 School of Mechanical Medical and Process Engineering, Faculty of Engineering, Queensland University of Technology, Gardens Point Campus, 2 George St., Brisbane, QLD 4000, Australia; l.alzubaidi@qut.edu.au (L.A.); haider.alwzwazy@hdr.qut.edu.au (H.A.A.)

**Keywords:** capacitive micromachined ultrasonic transducers, commercial CMUT probes, commercial ultrasound, CMUT performance, handheld ultrasound, high-end CMUT probes, CMUT catheters, PZT probes, Butterfly IQ, 4G CMUT

## Abstract

An emerging alternative to conventional piezoelectric technologies, which continue to dominate the ultrasound medical imaging (US) market, is Capacitive Micromachined Ultrasonic Transducers (CMUTs). Ultrasound transducers based on this technology offer a wider frequency bandwidth, improved cost-effectiveness, miniaturized size and effective integration with electronics. These features have led to an increase in the commercialization of CMUTs in the last 10 years. We conducted a review to answer three main research questions: (1) What are the commercially available CMUT-based clinical sonographic devices in the medical imaging space? (2) What are the medical imaging applications of these devices? (3) What is the performance of the devices in these applications? We additionally reported on all the future work expressed by modern studies released in the past 2 years to predict the trend of development in future CMUT device developments and express gaps in current research. The search retrieved 19 commercially available sonographic CMUT products belonging to seven companies. Four of the products were clinically approved. Sonographic CMUT devices have established their niche in the medical US imaging market mainly through the Butterfly iQ and iQ+ for quick preliminary screening, emergency care in resource-limited settings, clinical training, teleguidance, and paramedical applications. There were no commercialized 3D CMUT probes.

## 1. Introduction

Ultrasound scanning (US) can image internal body structures non-invasively and in real-time [1]. This resulted in its universal spread in healthcare as an efficient screening tool for disease prevention and diagnosis and pregnancy monitoring [2,3]. Clinical US frequencies normally range between 2MHz and 18 MHz, where lower frequencies possess lower resolution yet deeper body penetration and vice versa [4]. Different types of US transducers are available, including linear, curvilinear, phased arrays, or minimally invasive endoscopic or intravascular arrays [5]. Each transducer configuration has different optimal applications [6]. For example, linear probes are typically used for superficial organs such as breast, thyroid, and musculoskeletal examinations, curvilinear probes are typically used for abdominal and pelvic scanning, and phased array probes are typically used for cardiac imaging [6]. An alternative to the conventionally employed US transducers based on piezoelectric crystals (PZTs) are MEMS-based piezoelectric (PMUT) or capacitive (CMUT) micromachined ultrasonic transducers [5]. CMUTs were first manufactured by Stanford University in 1994 and commercialized by Hitachi in 2008 [7,8,9]. A CMUT consists of a micro-thin, metallized membrane suspended above a conductive silicon substrate by insulating posts. The suspended membrane and silicon substrate act as electrodes. Driving alternating electric current across the biased electrodes vibrates the membrane due to the change in capacitance, generating high levels of US waves, and vice versa [10]. Compared to PZTs, CMUTs allow for a broader frequency bandwidth, are more cost-efficient, have a miniature size, and are more convenient for mass production. Moreover, they are suitable for more complex configurations and geometries, thereby potentially covering all the previously mentioned imaging configurations using a single transducer [4,8]. Due to their miniature size, CMUTs can also be used in minimally invasive treatments, including catheter-based and endoscopic applications, and are considered ideal for wearable US technologies [11,12,13]. In comparison with PMUTs, they possess a higher bandwidth and resolution, making them more suited for medical imaging, particularly for high-frequency applications [5]. These advantages led to their rapid commercialization by several companies [7,8,9,14,15]. Previous reviews have explored the technical capabilities and research potential of CMUTs in US medical imaging [8,14]. However, limited literature has been published on the current commercial and clinical performance and applications of CMUTs in the space. In 2019, ref. [14] reviewed the advances in CMUT technologies and estimated a total of 23 companies offering CMUT products. Yole Development conducted a market analysis on US in 2018 and 2020 [7,9]: in their analysis reports, they listed and categorized 24 organizations that are engaged in the commercialization of CMUT devices intended for various medical and non-medical applications. In this paper, we consider companies that have directly commercialized CMUT sonographic devices for medical imaging applications. All the CMUT-based products developed by these companies along with their application focus in the market and their performance are reviewed. We also report on all the future work expressed by studies released in the past 2 years to predict the trend of development in future CMUT device developments and express gaps in current research. This review aimed to answer three main research questions: (1) What are the commercially available CMUT-based clinical sonographic devices in the medical imaging space? (2) What are the medical imaging applications of these devices? (3) What is the performance of the devices in these applications?

## 2. Methods

### 2.1. Pre-Search

The methods of this review were detailed and registered on the Open Science Framework (OSF) under the same title. This review is the first of its kind, pioneering an exhaustive analysis of commercial products utilizing a specific technology. It systematically assesses their performance against conventional alternatives and clinical modalities. We devised a customized review approach which was revised by a university health faculty library liaison to retain the systematic nature of the review throughout. Utilizing the findings reported in [14] and the market analysis conducted by Yole Development [7,9], we identified 8 companies that have commercialized CMUT sonographic devices for medical imaging applications, using the eligibility criteria discussed in the next section: Butterfly Network (Guilford, CT, USA), Kolo Medical (San Jose, CA, USA), Verasonics (Kirkland, Washington, United States), Vermon (Tours, Centre, France), Hitachi (Chiyoda City, Tokyo, Japan), Siemens (80333 Munich, Germany), Acoustoelectronics Laboratory (ACULAB) (Unversità degli Studi Roma Tre, Rome, Italy), and Philips (Eindhoven, High Tech Campus 5, Netherlands). ACULAB was included as the only organization identified in the market analysis as actively involved in the industrialization and commercialization of CMUT devices. Websites of each company were also browsed for CMUT products to be included along with the company name in the searching stage.

### 2.2. Search Strategy

**Figure 1:**
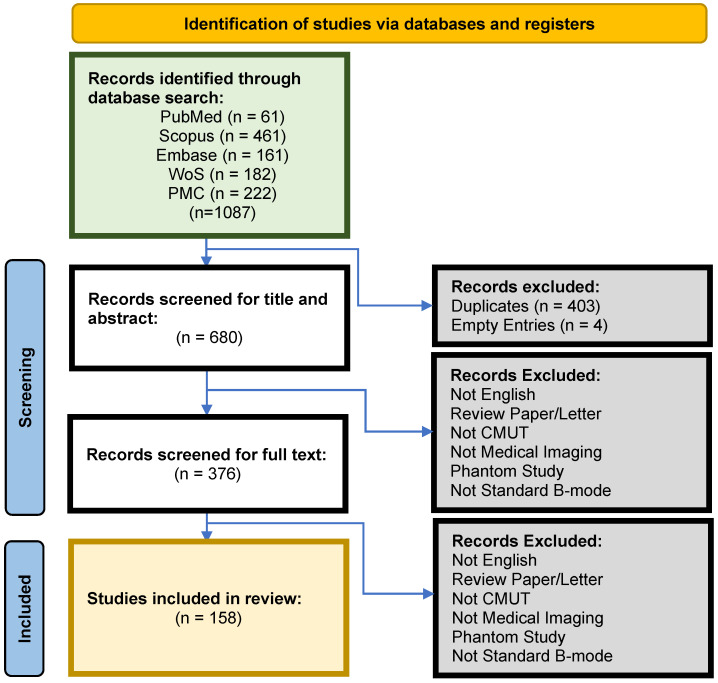
PRISMA methods for systematic study selection.

This systematic review was conducted in line with the PRISMA guidelines. Figure 1 illustrates the search protocol. The search strategy involved the 8 companies identified along with the products that are advertised on their websites. If the product was unnamed (e.g: CMUT Catheter for Therapy), it was not considered. Search strings were designed to capture the mention of the company in the context of CMUTs, the product in the context of the company, or the product in its own context. The main health databases were used: PubMed, Scopus, Embase, and Web of Science. The company products were also searched on PubMed Central (PMC) to uncover studies that have not referenced the company or product used in the main fields. The timeline considered was until 18 June 2024 with no field restrictions on all databases. The search queries used for each database were detailed in the OSF-registered methods. Records retrieved were exported from each database as a .ris or .nbib file and imported into Mendeley. The total number of identified records was 1087. After excluding duplicates, there were 680 articles remaining. The identified records were then further inspected to assess their relevance based on the criteria outlined in the eligibility criteria section. Titles and abstracts were screened based on the eligibility criteria. The full text of each study was screened for eligibility, and the final number of included studies in the review was 158.

### 2.3. Eligibility Criteria

This systematic review considered all the commercially available CMUT sonographic devices, probes, and catheters in the medical imaging domain. CMUT products used in studies were only deemed commercially available if they were developed by a specialized US company or organization. The scope of this review was limited to standard B-mode and Doppler-mode US modalities. CMUT products had to meet a minimum requirement of B-mode imaging capability, irrespective of their Doppler capabilities. Other US modes such as M- and A-mode were excluded. This review also only focused on standard US imaging, excluding other types such as photoacoustic and harmonic imaging, H-scan imaging, imaging with contrast agents, and high-intensity focused US. Included studies had to be either in vivo (human or veterinary) or cadaveric. This review was only concerned with clinical and pre-clinical CMUT performance, so phantom studies were excluded. In terms of article type, all studies were considered except for review articles and letters. Retracted articles were also excluded.

### 2.4. Data Extraction

Included studies were retrieved from Mendeley and imported into an Excel spreadsheet. For each paper, the title and citation, CMUT product utilized in the study, manufacturing company, application for which the product was employed, number of participants involved, and study design were extracted. Applications were based on the anatomy imaged and the objective of each study. The comparators, outcome measures, and corresponding performance results were recorded for evaluating the CMUT products. Additionally, information was extracted regarding the diagnosis and proposed procedure being investigated, as well as the anatomical organs and areas examined.

## 3. Results

### 3.1. Commercially Available CMUT Products

The search retrieved 19 commercially available CMUT products. No eligible Phillips products were found, so the company was excluded. There is a total of four clinically approved CMUT-based sonographic devices that are available in the market, produced by Butterfly Network and Hitachi. Hitachi was the first to introduce CMUT probes into the market with the Mappie system for breast cancer detection [16]. The product was later discontinued. In 2020, Hitachi commercialized the 4G SML44 CMUT probe. The company has experienced several mergers, splits, and acquisitions since its foundation in 1910. Most recently, their diagnostic imaging-related businesses were acquired by Fujifilm in 2021. Since, to the authors’ knowledge, no CMUT-based products were developed by Fujifilm since the acquisition, both Hitachi and Fujifilm were referred to in this review as one company using the original name, Hitachi. According to [16], the 4G CMUT probe is a single-probe solution that possesses an ultrawide-frequency bandwidth which is unachievable using conventional PZT probes and competes with high-end devices on high spatial resolution and sensitivity when imaging deep structures. Butterfly Network released a handheld CMUT probe called the Butterfly iQ (BiQ) in 2018 and then upgraded the probe to the Butterfly iQ+ (BiQ+) in 2020 [14]. Upgrades included shrinking the probe length and head, 60% higher frequency of pulse repetition, and 15% faster frame rates. The product was FDA-approved for 13 medical use cases and is composed of a 9000-element 2D CMUT array [14]. The device is a cost-effective single-probe solution, meaning that a single probe can emulate linear, curvilinear, and phased array configurations to image various body parts. The probe is coupled with a smartphone instead of a US system and features cloud-based services [14]. In February 2024, Butterfly Network launched its third-generation clinical US probe, the BiQ3, which won Best Medical Technology at the 2024 Prix Galien Awards (USA). With 2x the processing power of its predecessor, the BiQ+, the BiQ3 probe features enhanced image quality, frame rates, and frequency. Apart from ergonomic changes to probe design, size, and weight distribution to improve intercostal access in cardiac and pulmonary imaging and alleviate user strain, the BiQ3 introduces preliminary 3D imaging capabilities to HHUS through two novel presets: iQ Fan and iQ Slice. Over a region of interest, iQ Fan conducts continuous, real-time, bi-directional sweeps (±20°), whereas iQ Slice performs a single volumetric sweep, acquiring multiple slices of the region. However, both modes have their limitations, while the former preset is limited to pulmonary imaging, the latter mode is primarily for abdominal applications, with some pelvic and cardiovascular imaging. Despite extensive efforts detailed in the methods section, our review retrieved no studies on the BiQ3, likely due to its recent release. Kolo Medical has released six linear US probes (L62-38, L38-22, L30-14, M17-4, L22-8v, and L38-33v), two of which were commercialized in collaboration with Verasonics and based on the SiliconWave™ technology [8,17,18]. All their clinical CMUT sonographic devices are high/ultrahigh-frequency probes for high-resolution-related fields such as dermatology, ophthalmology, rheumatology, and musculoskeletal applications [18]. The rest of the companies, which include Vermon, Siemens, and ACULAB, have developed CMUT prototypes with specifications that are usually customizable based on the client’s research needs. ACULAB is also currently engaged in the development of volumetric imaging CMUT probes [19]. Specifications and details of all the commercially available CMUT products, at the clinical- and research-level, are presented in Table 1. Extracted raw results of review data can be found under Supplementary Materials.

**Figure 2:**
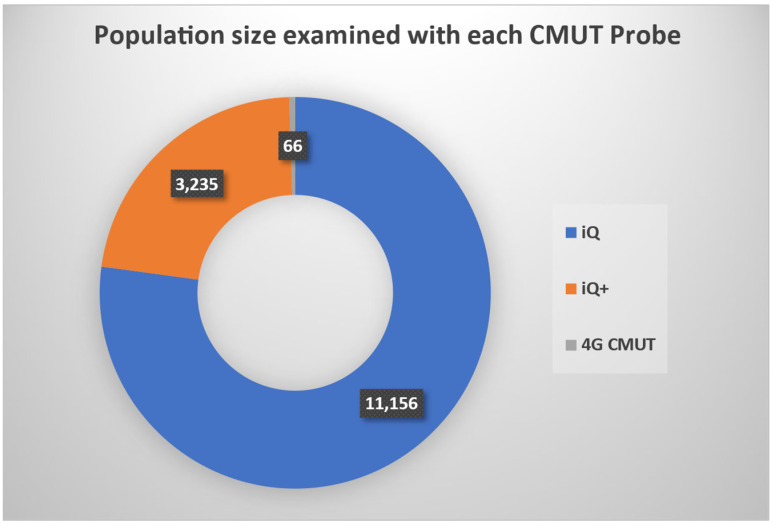
Population size examined with each CMUT probe: BiQ, BiQ+, and 4G CMUT. Probes that are not visible in the chart did not report the scanned population size in their studies or had a relatively negligible sample size.

Notably, 99.5% of the total population size in the CMUT studies retrieved were imaged using the BiQ or BiQ+, as shown in Figure 2. Remaining probes imaged around a single patient each.

### 3.2. Applications and Performance

Medical applications in this review were categorized into thoracic, cardiovascular, abdominal and pelvic, neuromusculoskeletal, and general imaging US. Thoracic US involved the breast, chest, and/or neck region. Studies that did not focus on one of the above US applications were classified as general imaging.

In terms of performance, this review presents the key performance findings reported by the reviewed studies on commercial CMUT devices. Quantitative standardization and meta-analysis were outside the scope of this review. The performance of the devices was reported relative to one or more competitors whenever available in the studies. Comparators were either in the form of other medical imaging modalities, US devices, diagnostic techniques, or the same CMUT device in a different setting. The performance was reported in terms of metrics based on outcome measures specified by the reviewed studies. For each measure, quantitative performance data were prioritized, and when unavailable or impracticable to report in detail, a qualitative summary was provided. Reviewed studies sometimes reported results along with the statistical significance of findings (*p*-values (*p*)) and precision of the estimate (confidence intervals (CI)). *p* indicates the probability of observing a given result if no real effect exists, whereas the CI shows the range of plausible values for the estimated parameter. A *p* < 0.05 suggests that the observed result is statistically significant and unlikely to have occurred by chance. CI is generally selected at 95%, meaning that 95% of the population mean will fall between the upper and lower limits.

We classified the outcome measures into the following categories: Diagnostic Performance, Correlation, Image Quality, Learning Experience, and Satisfaction/Feasibility.

- **Diagnostic Performance**: a measure of the performance or accuracy of a diagnostic tool or method in detecting a condition. The performance metrics of this measure included the following:
  ∘ Sensitivity (Se): the proportion of true positives among all patients with a condition/disease.
    ▪ $\mathrm{Se}=\frac{\mathrm{TP}}{\mathrm{TP}+\mathrm{FN}}$
      where TP = True Positives and FN = False Negatives;
  ∘ Specificity (Sp): proportion of true negatives among all patients without a condition.
    ▪ $\mathrm{Sp}=\frac{\mathrm{TN}}{\mathrm{TN}+\mathrm{FP}}$
      where TN = True Negatives and FP = False Positives;
  ∘ Diagnostic Accuracy (DA): overall proportion of correctly identified cases, both true positives and true negatives. The general formula for DA is as follows:
    ▪ $\mathrm{DA}=\frac{\mathrm{TP}+\mathrm{TN}}{\mathrm{TP}+\mathrm{TN}+\mathrm{FP}+\mathrm{FN}}$
  ∘ Positive Predictive Value (PPV): probability that a condition tested positive exists.
    ▪ $\mathrm{PPV}=\frac{\mathrm{TP}}{\mathrm{TP}+\mathrm{FP}}$
  ∘ Negative Predictive Value (NPV): probability that a condition tested negative does not exist.
    ▪ $\mathrm{NPV}=\frac{\mathrm{TN}}{\mathrm{TN}+\mathrm{FN}}$
  ∘ Area Under the Curve (AUC): area underneath the Receiver Operating Curve (ROC), which is a graphical representation of the true positive rate versus the false negative rate where their sum is equal to one, and they range from [0,1] and [0,1], respectively. AUC is ideally equal to 1 and at least larger than 0.5;
  ∘ Diagnostic Duration (Time): time taken to complete a diagnostic procedure or obtain results;
  ∘ Feasibility (Yes/No): determines whether a diagnostic method is practical and can be implemented effectively in clinical settings;
  ∘ Cannulation or Injection Accuracy: rate or accuracy of successful cannulation or injection relying on a diagnostic tool/method for guidance;
- **Correlation**: a measure of the degree of similarity or agreement between test results and a reference standard, or the consistency between different observers and test conditions. Performance metrics included the following:
  ∘ Inter-observer and Intra-observer Agreement (Kappa Agreement (k), ICC): this entailed two categories of agreement/reliability—agreement among different observers/operators/raters (interrater) over an identical exam and consistency of a single rater across repeated exams (intrarater);
    ▪ Kappa Agreement/Cohen’s Kappa (k): agreement between two different raters on categorical assessments. It ranges from −1 (complete disagreement) to +1 (complete agreement), with 0 indicating random chance agreement. Standard k is usually employed for nominal categorical assessments, while variants are adapted for ordinal data or assessments by more than two raters;
    ▪ Intraclass Correlation Coefficient (ICC): reliability or consistency across two or more different raters on continuous measurements. This metric was also employed by studies for intrarater reliability. It typically ranges from 0 (no reliability) to 1 (perfect reliability);
  ∘ Correlation with a reference standard (Pearson’s Correlation/Spearman’s Rank Correlation):
    ▪ Pearson’s Correlation (r): measures linear relationship between two continuous assessment variables;
    ▪ Spearman’s Rank Correlation: measures correlation of the rank of two variables. It indicates the degree to which the variables are monotonically related, even if their relationship is not linear;
  ∘ Measurement Variability/Reproducibility: fluctuation of results of tests repeated under similar conditions.

The most common interpretation of correlation values is as follows:

- Values ≤ 0: no or poor agreement;
- Values 0.01–0.20: poor or slight agreement;
- Values 0.21–0.40: fair agreement;
- Values 0.41–0.60: moderate agreement;
- Values 0.61–0.80: substantial agreement;
- Values 0.81–1.00: almost perfect agreement;
- **Image Quality**: a measure of the clarity, resolution, and usefulness of images produced by diagnostic tools.
  ∘ Image Resolution: spatial (e.g., axial and lateral) technical image details;
  ∘ Image Clarity: delineation of structure, sharpness and presence of artefacts;
  ∘ Interpretability: images deemed useful for diagnosis;
- **Learning Experience**: a measure of improvements in US training, knowledge, interpretation and technical skills related to diagnostic procedures, often before and after training or experience. Metrics included the following:
  ∘ Skill Acquisition and Retention (Objective Structured Clinical Exams (OSCE), Exam Scores, Theoretical/Practical Tests): assesses how well trainees acquire and retain US skills over time, often through examination;
  ∘ US and Anatomical Knowledge Improvement (Exam Scores): assesses, often through exams, the improvement in anatomical or US-specific knowledge after training;
  ∘ Training Effectiveness (Experts vs. Novices): assesses the performance of a trainee post-training against experts;
  ∘ Feasibility of Teleguided Learning: assesses the feasibility or performance of a trainee or a novice in conducting US examination and/or interpreting US findings with teleguidance;
- **Satisfaction:** a measure of user confidence, ease of use, and operational efficiency. Metrics included the following:
  ∘ User Satisfaction Scores (Surveys, User Feedback): evaluates subjective satisfaction in using the US tool, often through surveys or user feedback;
  ∘ Confidence in Self-Assessment (Confidence Percentage, Surveys): measures how well users feel they performed.

**Figure 3:**
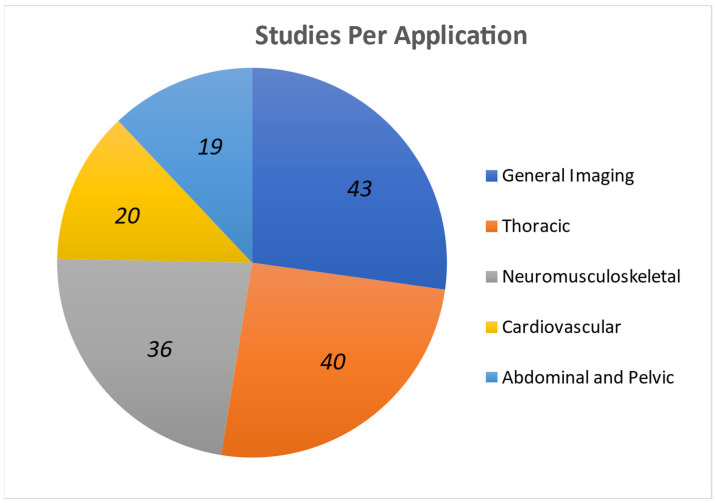
Applications and outcome measure statistics: number of studies that focused on each application.

As shown in Figure 3, a significant proportion of the studies were dedicated to thoracic, general imaging, and neuromusculoskeletal applications. Studies were categorized as prospective (POS) or retrospective observational studies (ROS), cross-sectional studies (C-S), randomized control trials (RCT), case studies (CS), feasibility studies (FS), cadaveric studies (CAD), or veterinary studies (VET). Diagnostic Performance was the most employed outcome measure in terms of both number of studies and population size. Furthermore, as indicated in Table 2, the majority of the patients were studied prospectively, specifically 71.4%. Only 1.6% of the population size examined were cadaveric and veterinary.

#### 3.2.1. Thoracic

Whereas thoracic applications in this review involved the neck, breast, and lung regions, approximately 25% of studies were for pulmonary applications, making the lung the most examined structure. Table 3 details the performance of the CMUT products in thoracic applications. BiQ and BiQ+ were the only CMUT medical imaging devices used for pulmonary applications, where the former device was used in 88% of the studies, and it was a reference among Handheld US (HHUS) devices for lung US (LUS) and Emergency Room (ER) applications. Only four of the pulmonary studies were not COVID-19 related. Apart from availability, interest in the BiQ for COVID-19 applications is possibly due to the device’s ease of decontamination [30]. The device facilitated clinical assessments and decision-making for patients with COVID-19 of various severity presentations [31]. In many studies, it was able to identify, detect, and/or assess COVID-19-related pathologies and malaria cases [32], pulmonary oedema [32], post-acute sequelae (PASC) [33], and pneumonia and pneumothorax [31]. A retrospective study on 100 patients found that the BiQ could accurately assess moderate-to-high-severity COVID-19-related pathologies with 92% diagnostic accuracy [34]. Ref. [31] also retrospectively reported that LUS scores of 18 COVID-19 patients perfectly correlated (Pearson Correlation (PC) = 0.99) with a high-end US device, Venue Go. (GE Healthcare, Chicago, IL, USA). Since chest CT is considered the gold standard for diagnosing lung pathologies [35], the BiQ was compared against chest CT and chest X-ray in nine studies. Ref. [36] compared chest CT with the BiQ in diagnosing 51 COVID-19 patients. The BiQ was 100% sensitive and 78.6% specific, highly correlating with chest CT with an ICC of 0.8. With chest X-ray, ref. [37] found a significant association (*p* = 0.034) between the decision-making for hospital referral using the BiQ and chest-ray based on a severity scale they constructed. The study concluded that the device could reduce uncertainty in moderate cases, facilitate prompt referral, and prevent unnecessary referral [37]. The BiQ also showed versatility in various thoracic settings, as it was found suitable for teaching LUS virtually and in person and for telemedicine with home-isolated COVID-19 patients [38,39,40,41]. It was easy to use and learn with adequate interobserver correlation for discerning abnormal LUS scans in 13 patients [42]; and when comparing 44 teleguided patients who were self-imaging their lungs against US experts’ results, there was an expert agreement of 87% and k = 0.49 [38]. Moreover, 98% of the patients felt confident in self-examination.

#### 3.2.2. Cardiovascular

The cardiovascular performance of the commercial CMUT probes is reported in Table 4. Around 13% of the CMUT studies were cardiovascular, less than 1% of which were neither BiQ nor BiQ+ related. Cardiovascular structures imaged using CMUTs in the literature included the heart [72], carotid artery [73,74], radial and ulnar arteries [75], internal jugular vein (IJV) [74,76,77], and inferior vena cava (IVC) [77]. The literature reports on the feasibility of the device for guiding cannulation in such structures [78]. The device significantly outperformed high-end US systems, such as the Mindray TE7 (Mindray North America, Mahwah, NJ, USA), for first-pass peripheral intravenous attempts (92.59% vs. 68.75%) when performed by emergency physicians on 59 patients with a history of difficult-to-obtain peripheral intravenous (PIV) access [79]. Two studies conducted on 113 patients in total showed that the BiQ is reliable for assessing the intravascular volume status to manage heart failure [74,76]. A US examination of the jugular venous pulsation using the device could accurately estimate right atrial pressure in both obese and non-obese patients, outperforming visual and IVC collapsibility assessments for obese patients [74,76]. Other studies reported on diagnosing venous gas emboli in divers’ arterial circulation, where results obtained by the BiQ demonstrated moderate agreement with Vivid q™ (GE Healthcare, Chicago) and O’Dive™ (Azoth Systems, Ollioules) [80]. The BiQ produced fewer quality images with inferior sensitivity and specificity than the former device, yet, higher than the latter device [80]. Overall, it was concluded that the device was not a replacement for post-dive venous gas emboli assessment [80]. Ref. [20] also used the BiQ to diagnose various cardiac presentations in the ER, including respiratory failure, dyspnea, novel atrial fibrillation, cardiogenic shock, and hypoxia. The device was adequate for cardiac imaging and could generally acquire all cardiac views, address basic resource-limited settings and clinical questions and identify substantial pathology [20]. The quality of the cardiac images was lower compared to the other presets in the probe itself (e.g., abdominal and musculoskeletal) due to a dip in resolution and frame rate, especially when Color Doppler was used [20]. The BiQ and most other HHUs lack advanced echocardiographic applications and spectral Doppler capabilities, rendering them less suitable for quantitative assessments [20]. In terms of US education, due to the absence of established cardiac POCUS training protocols in medical schools [81], the BiQ and BiQ+ were used as an educational or training tool for that purpose in four of the studies. Ref. [81] found that 54 medical students showed high satisfaction with the BiQ device. However, they struggled to learn US acquisitions through the PLAX, PSAX, and A4C views [81].

#### 3.2.3. Abdominal and Pelvic

Abdominal and pelvic studies (reported in Table 5) were 12% of the total number of papers retrieved. CMUT-based devices, and in particular the BiQ, have been used to image numerous abdominal organs, including the kidney, gallbladder [91], liver [24], spleen [6], and pelvis [92], as well as to examine the blood vessels that connect to some of these organs such as the aorta [93]. The BiQ was employed in large-scale hospital environments for abdominal imaging in four studies [20,91,93,94]. The overall performance of the BiQ in investigating causes of abdominal and obstetric presentations, ranging in degrees of severity, was found to be sufficient to resolve clinical questions. The device’s performance was also found to be outstanding in terms of image quality, particularly compared to its other modes such as cardiac imaging. The device outperformed clinical assessment for diagnosing and managing 19 patients presenting with kidney disease, confirming all findings and determining new ones in 30% of the cases in one instance [92] and determining 50% new findings in another study [95]. Sonographers referred to the images acquired by the CMUT device in the latter study as “stunning images” [95]. In terms of prostatic disease, the BiQ assessments correlated better against guideline imaging than other HHUS, such as the Clarius C3 (Virtual Way, Vancouver, Canada), in assessing the prostate gland volume of 78 patients in an RCT study (ICC 0.78 vs. 0.71), and it was found more reliable [96]. Studies also report on the feasibility of using the BiQ for monitoring pregnancy [94] and heart rate in newborns [97]. For vascular imaging in the abdomen, physicians from the Prince Sultan Military Medical City Hospital in Saudi Arabia compared the measurement reproducibility of the BiQ with a conventional US device (CUD), the EPIQ 7 (Philips, Bothell, WA, USA), on 114 participants for large-scale abdominal aortic aneurysm (AAA) screening [93]. Inter- and intra-operator reproducibility of aortic measurements correlated nearly perfectly (ICC > 0.8), except for the proximal location, with no struggle due to diminished spatial resolution or image acquisition [93]. The study concluded that the BiQ is an inexpensive, non-inferior alternative for AAA screening [93]. Similarly, a prospective protocol study was conducted using the BiQ+ on 1000 hospitalized patients presenting a picture of a common acute infection. The device was recommended to assess the presence of hydronephrosis as well as expedite the diagnosis [98]. Learning abdominal US using the BiQ also was not found challenging as 194 novices attained 96.4% diagnostically interpretable images for obstetric applications after only three hours of training [94]. A similar case was reported for the evaluation of the bladder (90% confidence level) and other pelvic structures (100% diagnostic accuracy) [99]. The reason for the ease of adoption and the outstanding performance of the BiQ in abdominal and pelvic applications is that while the depths of the abdominal organs are large and usually require a low-frequency curvilinear probe [100] (which is not the BiQ’s strength), the organs in this region are often large and may be easier to image compared to other regions.

#### 3.2.4. Neuromusculoskeletal

Neuromusculoskeletal (NMSK) US can accurately expedite the identification of distinctive features of arthritic diseases and is considered to be nearing, if not on par with, MRI in imaging soft tissue injuries [111]. Approximately 23% of the CMUT studies found were NMSK-focused. For the overall performance of a commercial CMUT device in NMSK clinical applications other than the BiQ, three MSK radiologists retrospectively and independently compared the US exams acquired using the Hitachi 4G CMUT probe and a traditional L64 PZT probe (Hitachi Ltd., Chiyoda City, Tokyo, Japan) on an Arietta 850 workstation (Hitachi Ltd., Chiyoda City, Tokyo, Japan). The exams were for 66 patients with various MSK diseases [112]. Nearly one-third of the regions examined were shoulder-related [112]. The cases included the following: acute supraspinatus tendon tear, vastus lateralis muscle strain, entrapment neuropathy in the radial nerve, Dupuytren’s contracture, Ledderhose disease, Epicondylitis, and Acute De Quervain tenosynovitis [112]. There was a close correlation between the diagnostic performance of both probes [112]. Compared to the CUD, the 4G CMUT had better image panoramicity (width) and deep structure definition but poorer image quality in superficial tissue evaluations and the Doppler signal [112]. The authors concluded that improvements regarding these limitations must be made before the device could efficiently replace traditional PZT probes for this application [112]. As for the BiQ, physicians from an outpatient clinic compared the device with a CUD, HS40 (Samsung, Seoul, Seoul-t’ukpyolsi, Republic of Korea), by scanning 32 patients, at least one of whose joints were swollen and tender in order to assess the HHUS performance in inflammatory arthritis (IA) patients [113]. The study involved four different types of arthritis: rheumatoid, psoriatic, gouty, and lupus [113]. Images from both devices correlated nearly perfectly in B-mode (97%). The BiQ had a similar examination time to the CUD device, but it failed completely in detecting any Power Doppler (PD) signal in PD mode [113]. It was concluded that the B-mode BiQ was feasible and accurate for evaluating structural joint damage and inflammation in IA patients, but PD mode necessitates developments before it is viable for blood flow detection [113]. BiQ also had a similar performance to ophthalmic CUDs in ocular imaging [114,115]; it correlated well with arthroscopic findings [116], and it was even more sensitive than fluoroscopy [117,118] for ankle examinations on cadavers. However, it was less reliable than arthroscopy in distinguishing the different injury stages through sagittal translation in syndesmosis instability [116]. Moreover, BiQ proved to be viable for assessing and assisting in treating brain aneurysms (100% surgery success rate) in human and cadaveric brains [119]. In the arm, ref. [120] readily determined the nerve involvement and enlargement in eight patients with leprosy by imaging their wrist, forearm, elbow, and mid-humerus to visualize the bilateral median, ulnar, C5 root, and greater auricular nerves with a BiQ [120]. They compared it against a GE Logiq E (GE Healthcare, Chicago, IL, USA), and despite the BiQ having one-third of the frequency bandwidth than that of the CUD, the cross-sectional areas measured with both devices correlated (Pearson product-moment correlation = 0.73 (*p* < 0.001)). They concluded that the device may assist in the diagnosis of leprosy in areas with limited healthcare resources because of the portability and low-cost nature of such devices [120]. An RCT conducted by [6] compared the BiQ with multiple CUDs on superficial structures of 29 patients and found no distinction in the diagnostic qualities of the images. Such diagnostic quality can be very useful in cases such as the ones discussed in [20] where, in a presentation of submandibular swelling of the jaw, the authors were able to identify a submandibular abscess using the BiQ. As for joints, ref. [121] proposed a protocol to scan hand, wrist, elbow, and shoulder joints using the BiQ for early detection of arthritis. They found that the course based on this protocol was fit for training dermatologists without pre-requisite knowledge [121]. Finally, numerous studies showed that the BiQ is very effective and easily taught by experts and student tutors alike [122] for NMSK US teleguided learning and even self-scanning [121,123]. Further performance details are shown in Table 6.

#### 3.2.5. General Imaging

Studies focusing on two or more of the specified US applications were categorized as general imaging. General imaging involves two very commonly employed exams: Rapid US for Shock and Hypotension (RUSH) and Focused Assessment with Sonography for Trauma (FAST). Both comprise examining structures in the lungs, heart, thorax, abdomen, and pelvis [146]. Ref. [147] compared the BiQ with the conventionally used US system, Phillips Sparq (Phillips, Amsterdam, The Netherlands), in the ER on 50 healthy adults by conducting RUSH exams and found that the images did not differ in image acquisition latencies or image quality, concluding that the device may be an alternative for RUSH exams. Emergency physicians in another study also performed FAST exams on 29 patients in an RCT, comparing the BiQ against a GE LOGIQ S7 (GE Healthcare, Chicago, IL, USA) with three different transducers: SP-D (GE Healthcare, Chicago, IL, USA), C1-5-D (GE Healthcare, Chicago, IL, USA), and ML5-15 (GE Healthcare, Chicago, IL, USA), by scanning several body regions while switching between imaging pre-sets and transducers accordingly [6]. The process is also known as US hypotension protocols (UHPs) [6]. The time taken by the BiQ to perform the UHPs was significantly shorter than the CUD, by approximately 2 whole minutes (4.08 min vs. 5.8 min), and the number of diagnostic quality images did not differ between the two devices [6]. Ref. [148] also compared the image resolution, detail, and quality of the BiQ against the Sonosite M-turbo in an RCT on 74 patients using three blinded sonographers. Imaging purposes included the spine, transversus abdominis plane (TAP), and diagnostic obstetrics [148]. The BiQ was found to be superior in all image categories with the spine and similar in resolution and image quality in TAP images yet inferior in all image categories in obstetrical images [148]. The study concluded that the BiQ may be a POCUS alternative to the expensive machine; however, it is better suited for aesthetic rather than diagnostic obstetrical indications. To compare how the BiQ fares against a HHUS, 24 US experts compared the BiQ against three other HHUS, including the Vscan Air™ (GE Healthcare), the Lumify™ (Philips Healthcare) and the Kosmos™ (EchoNous, Inc.) systems, by examining patients through three POCUS views (FAST right upper quadrant view, transverse view of the neck with the IJV and carotid artery, and cardiac parasternal long-axis view) [149]. While BiQ ranked the second highest in ease of use, it was the worst by a large margin in terms of image quality and was indicated as the least likely to be purchased by the experts [149]. Two out of the three POCUS examinations were conducted with a cardiac preset, one of which relied on Color Doppler. Similarly, ref. [150] preferred the BiQ over Lumify™ (Philips Healthcare) in their educational prospective study for imaging deep and superficial structures but not for imaging the heart, lung, and abdomen. These studies may imply that the BiQ performs the poorest in cardiovascular imaging applications, particularly the ones involving Doppler capabilities, likely due to a poor phased array arrangement and its blood-flow monitoring for its weak Color Doppler features [100]. Details of the performance of the CMUT probes in general imaging applications are shown in Table 7.

### 3.3. Future Work of Modern Studies

Table 8 summarizes the future work reported by commercially available CMUT-related modern studies for the years 2023 and 2024. The trend focuses on expanding the range of clinical applications for CMUT-based HHUS, in particular BiQ and BiQ+, by validating the devices on larger patient populations in various settings, especially low-resource settings. The trend promotes improving training protocols to teach more medical practitioners, as well as non-practitioners, how to use the devices. Moreover, it promotes exploring the integration of AI to improve the devices’ utility. Only [142] has proposed interest in enhancing 3D imaging capabilities of CMUT-based HHUS devices.

## 4. Discussion

The review retrieved 158 studies and 19 standard medical imaging CMUT products, produced by seven US companies. Four clinically approved CMUT probes are currently available: the BiQ, BiQ+, BiQ3, and 4G CMUT probe. Hitachi and Butterfly Network shared a common interest in the single-probe CMUT capability, which was a characteristic exhibited by all three products. Hitachi was more engaged in developing a high-end CMUT device, while Butterfly Network focused on handheld US technologies. All other companies developed CMUT products for research purposes, primarily by having main CMUT products which they often adjust to manufacture tailored versions upon client request. Kolo Medical and Verasonics expressed interest in the high-frequency benefits of CMUTs, as evidenced by their exclusive production of high-frequency and ultrahigh frequency CMUT probes [18,182]. ACULAB’s exclusive area of focus centered on the utilization of reverse fabricated and dual probes, whereas Siemens prioritized exploring the potential of 3D imaging for CMUTs through the implementation of 2D arrays. Vermon was the only company manufacturing CMUT catheters.

With regards to applications and performance, ref. [14] postulated that CMUTs can replace PZT technologies in any medical US probe. This postulation is supported by retrieved results of this review, especially in instances where research-purposed CMUT probes were compared against their PZT counterparts and showed promising results [22,24,27,29,125]. Commercial products employing the CMUT technology have been used to image nearly every part of the body and for numerous applications and sub-applications, including pulmonary, cardiac, thoracic, endoscopic, vascular, ocular, cranial, musculoskeletal, nerve, and general imaging US. The devices have established their niche in the medical US imaging market mainly through the BiQ for quick preliminary screening, emergency care in resource-limited settings, clinical training, teleguidance, and paramedical applications, as reported by [20]. Ref. [20]’s study was the most pertinent to this review because it comprehensively evaluated the BiQ’s performance across diverse clinical applications and intercompared its presets, showing that the BiQ performed best in musculoskeletal and abdominal applications and was adequate, yet typically with relatively worse performance, for cardiac and blood-flow imaging (and in some applications not viable at all). The BiQ was an effective tool that offered good image quality in the majority of functions, especially when employed by experts [20], but it does not replace high-end US examinations as reported by [183].

In terms of limitations and future work, the literature has investigated the performance of the CMUT devices on approximately 14,462 human patients, which is considered a limited population size. Moreover, 92% of the studies were related to the BiQ and BiQ+, which are both handheld devices manufactured using the same technology. Generalizing the reviewed performance to CMUTs becomes challenging. A total of 25% of the studies were concerned with these products being used as training tools for medical students, suggesting that they may become the primary choice for education and general research US due to their teleguidance capabilities during self-isolation and being easy to learn [184]. No studies compared the relative performance of the BiQ, BiQ+, and BiQ3. In fact, no studies were retrieved on the performance of the BiQ3, possibly due to its very recent release. The only other clinical CMUT product, the 4G CMUT probe, was used to examine 66 patients in one application, which is not a sufficient sample size to generalize the performance of high-end CMUTs. Future studies shall examine a larger sample size and challenge the device in more applications and conditions. The high-frequency bandwidth advantage possessed by CMUTs suggests that they may excel in high-frequency applications, yet no clinical CMUT catheters were presently available. Their imminent development is anticipated through Vermon. There were also no commercialized dedicated 3D CMUT probes. Siemens and, according to [19], ACULAB are working towards them [26]. Future studies may also expand on the 3 BiQ3’s application-limited 3D imaging capabilities to design novel 3D US single-probe handheld devices. Despite the market focus of CMUTs being on wearable and handheld applications [5,11,185] and the technology’s ability to adopt complex array arrangements, configurations, and geometries, no commercially available wearable CMUTs existed to date, nor have any companies expressed interest in developing them. Furthermore, no studies have suggested such future work. We surmise that this advantage, along with portability, cost-efficiency, and frequency bandwidth, are the main reasons for CMUTs to replace PZTs. The medical imaging potential offered by CMUTs is substantial, and we expect the reviewed companies to continue discovering novel use cases and commercializing the technology at an even faster rate.

## 5. Conclusions

This review performed a systematic, exhaustive analysis of the currently available commercial CMUT systems in medical US imaging. Despite their recent introduction to the market in 2008, this review retrieved 19 devices, 4 of which were clinically approved. Available probes presented a promising alternative to the conventionally used PZT technology in the field, demonstrating comparable image quality and performance in many applications. The reviewed literature highlighted their distinct advantages, including wider frequency bandwidth, cost-effectiveness, miniaturized size, and integration capabilities with electronics. These qualities have encouraged their rapid commercialization, with emerging interest from US companies with large influence. Notably, the BiQ and BiQ+ have gained recognition for their suitability in quick preliminary screenings, emergency care in resource-limited settings, clinical training, and paramedical applications. The probes did not only establish themselves in diverse clinical applications but also in medical education and research. The teleguidance capabilities of these devices proved invaluable in self-isolation and remote learning settings. While the probes excelled in musculoskeletal and abdominal applications, their performance in cardiac and blood-flow imaging displayed some limitations. This review discovered limitations such as the relatively limited sample size of patients studied and the need for further research in various applications and conditions, especially for the 4G CMUT probe to investigate the technology’s performance in high-end US imaging. The potential for 3D US and wearable, distributed device imaging remains unexplored. Significant strides were made, and are being made, in commercial CMUT devices, and as these large companies continue to explore novel use cases of the technology, advancements in the field of US medical imaging are anticipated.

## Figures and Tables

**Table 1:** Details of all the commercially available products.

Company	Probe	Price	Center Frequency	Transducer Type	Portability	Application	Features	References
Clinical								
**Hitachi**	Mappie	No longer available	10 MHz		US Platform-Compatible Probe	Detecting Breast Cancer		[16]
	4G CMUT probe (SML44)		11 MHz	Adjustable	US Platform-Compatible Single-Probe	General Imaging	-High Doppler and Color Doppler sensitivity -High-resolution imaging of deep structures	
**Butterfly Network**	iQ, iQ+	USD 2999	5 MHz	Adjustable	Handheld Single-Probe	General Imaging	-Cloud Storage -AI guidance for user training	[20]
	iQ3	USD 3899	6 MHz				-Cloud storage -3D imaging capabilities -AI guidance for user training	
**Research**								
**Kolo Medical**	L62-38		50 MHz	Linear	US Platform-Compatible	Ultrahigh resolution for dermatology, ophthalmology, and rheumatology	Ultrahigh/high frequency US	[21]
	L38-22		30 MHz			Superficial imaging in Dermatology and MSK		[8,17]
	L30-14		22 MHz			Neonatal, Paediatric, and MSK applications		
	M17-4		8–10 MHz			General imaging		
**Kolo Medical + Verasonics**	L22-8v		15 MHz			MSK and small parts imaging		[18,22]
	L38-33v		30 MHz			Pre-clinical and superficial imaging		
**Vermon**	CMUT Catheter for Therapy (Interstitial CMUT)		6 MHz	Catheter	US Platform-Compatible Probe US	IVUS + tumor ablation and interstitial surgery	Compatible with MRI guidance	[23]
	128-element array probe		5 MHz	Linear		Imaging and characterization of liver tissue		[24]
	CMUT Intracardiac imaging catheter (CMUT-based 1D ICE)		7.5 MHz	Catheter		Intracardiac echocardiography (ICE)	Integrated with an ASIC chip	[23]
	Dual-Mode CMUT probe		1 M or 15–20 MHz	Linear		Diagnosis and targeted therapy		[25]
**Siemens**	9 MHz linear array		9 MHz	Linear		Neuromusculoskeletal	Electronically Scanned 3D imaging	[26]
	3.5 MHz curved array		3.5 MHz	Curved		Abdominal Imaging	40 mm radius of curvature	[27]
**ACULAB**	HF12 CMUT Probe		13.6 MHz	Linear		Imaging of the carotid artery	High frequency and small probe head	[28]
	Reverse fabricated CMUT Probe		12 MHz	Linear		Vascular, small parts, rheumatology, and anesthesiology imaging	Multichannel analogue front-end electronic circuits housing	[29]

**Table 2:** Patient population size (n) per study design.

Study Design	POS	C-S	RCT	FS	ROS	CS	VET	CAD
**n**	10,326	1554	1679	453	134	82	170	64

**Table 3:** Performance of commercially available medical CMUT devices in thoracic US imaging applications.

Cite	n	Comparator	Outcome Measure	Performance
[41]	3	Symptomatic Improvement	Diagnostic Performance	US findings lagged behind symptomatic improvements
[30]	-	-	-	No conclusive result
[42]	13	Interobserver	Correlation	Substantial interobserver correlation in identifying abnormal US scans; moderate correlation for COVID-19 manifestations (except consolidations and pleural thickening/effusions)
[37]	61	Chest X-ray	Correlation	Significant association of LUS severity scale with appropriate referral (*p* = 0.001); with chest X-ray (*p* = 0.034)
[40]	1	Clinical Diagnosis	Diagnostic Performance	Confirmed clinical findings and guided therapy by predicting patient outcomes
[43]	21	-	-	Guided placement of pulmonary, carotid, and femoral artery catheters
[38]	44	Expert vs. Non-expert	Correlation, Learning Experience	Expert agreement: 87%; Kappa agreement of 0.49 for LUS results; 98% of participants were confident in self-exam
[39]	1	Chest Radiography	Correlation	Detected findings unseen in radiography, correlated with clinical diagnosis
[22]	-	High-Frequency (10 MHz) PZT Probe	Image Quality	Improved spatial (axial and lateral) resolution compared to conventional probes
[44]	-	-	Learning Experience	No conclusive result
[36]	51	-	Diagnostic Performance	Se = 100.0%, Sp = 78.6%
[45]	118	DUS Offline vs. Bedside	Correlation	Mean absolute variability = 4.2% (95% CI: 2.8–5.6%); moderate correlation
[46]	202	12, 8, and 6 Zone LUS for COVID-19	Diagnostic Performance	6-zone LUS was the best screening tool (Se = 94.1%, Sp = 83.5%); 12-zone LUS had the highest Sp (Sp = 92.3%)
[47]	1	VFX13-5 Probe vs. Conventional Probe	Image Quality	Resolution improved at 5 MHz (*p* < 0.05); elevated side lobe artifacts were observed at 8 MHz.
[31]	18	Standard High-end US (Venue GO)	Correlation	No statistically significant difference between LUS scores
[34]	100	POCUS vs. Clinical Outcomes	Diagnostic Performance	92% accuracy in diagnosing COVID-19; good discriminatory performance for mortality and critical care admission (AUC = 0.80, 0.80, 0.82); mortality risk: 2.5% (lowest quartile) vs. 42.9% (highest quartile)
[48]	393	HOME-CoV and 4C mortality score	-	No conclusive result
[49]	1	N/A	-	No conclusive result
[50]	228	RT-PCR	Diagnostic Performance	Se = 94.4%, Sp = 95%; higher sensitivity than RT-PCR (80%)
[51]	-	Computed Tomographic Angiography (CTA)	Correlation	Correlated with CTA output. Feasible for pre-operative perforators mapping in DIEP flap breast reconstruction
[52]	170	Standard of Care	Diagnostic Performance, Correlation	Se = 97%, Sp = 100%, and 97.6% agreement with Standard of Care (κ = 0.95, *p* < 0.0001); 100% detection rate for sonographically visible cancers
[53]	52	ML vs. Radiologist Assessment	Correlation	Perfect correlation
[54]	20	N/A	Diagnostic Performance	rPTX successfully identified and imaging is useful within 4 h from TT-removal
[55]	-	US NT vs. Landmark Technique	Diagnostic Performance	Accuracy: 67% vs. 85% (*p* = 0.019); time: 19.9 s vs. 10.7 s (*p* = 0.001)
[56]	115	Novice with BiQ vs. Expert with High-end US	Diagnostic Performance, Correlation	Se = 100%; Sp = 86%; Cohen’s κ = 0.73 (95% CI: 0.57–0.9, *p* < 0.0001)
[57]	-	Teleguidance vs. Traditional Teaching	Learning Experience, Satisfaction	No difference in performance and similar satisfaction
[58]	110	Chest X-ray with and without Atelectasis	Diagnostic Performance	Se = 100%, and Sp = 13.5% (95% CI); with Atelectasis: Se = 67.2%, and Sp = 29.7%; without Atelectasis: Se = 56.3%, and Sp = 43.2%
[59]	28	Chest CT	Diagnostic Performance	High Se and lower diagnostic efficacy for mild–moderate disease: not an alternative for CT in COVID-19 assessment
[60]	3	Chest X-ray	-	No conclusive result
[61]	21	Expert Review	Image Quality, Learning Experience	Only 5.9% of 728 zones were low quality; longer scanning time (>7 min) improved accuracy
[62]	40	Chest X-ray	Diagnostic Performance	AUC = 89.2% (95% CI: 75.0–100%; *p* < 0.001); Se = 93.3%, and Sp = 80% (*p* < 0.001)
[63]	30	Medically untrained vs. POCUS Experts	Image Quality	Novices acquired interpretable, expert-quality LUS clips at home with minimal training (median 7 out of 8 zones, IQR 6–8; *p* = 0.42)
[64]	134	Clinical Findings	Diagnostic Performance	Se = 45.5%, Sp = 77.3%, AUC = 63.9% vs. Se = 72.7%, Sp = 79.8%, and AUC = 80.3%
[65]	96	Chest X-ray	Diagnostic Performance, Correlation	Se = 71.7% vs. 62.2%, Sp = 65.1% vs. 71.4%, and the presence of bilateral B-lines showed the greatest likelihood ratio for accurately identifying COVID-19
[66]	1	-	-	No conclusive result
[67]	31	-	Image Quality, Diagnostic Performance	90% of images were of good quality after 2.5 h of training; 5 min per exam; B-lines were significantly associated with weight gain, a clinical marker of worsening heart failure
[68]	100	Chest X-ray	Diagnostic Performance, Correlation	Se = 98.51% (95% CI 91.96–99.96%), Sp = 87.9% (95% CI 71.8–96.6%), DA = 95% (PPV = 94.3%, NPV = 96.7%), 30% scans were normal (97% correlation with normal Chest X-ray), and 70% scans were abnormal (90% correlation with abnormal Chest X-ray)
[69]	754	Chest Radiography	Diagnostic Performance	Diagnosed 96.6% of pneumothorax cases on initial imaging, comparable to chest radiography. Only one case was missed by US. Chest radiography use decreased from 98.2% to 25.8% after US introduction. Increased confirmatory investigations post-US compared to chest radiography
[70]	18	Expert vs. Non-clinician	Correlation, Satisfaction	96% of the 1339 scans were deemed interpretable (k = 0.67) | 100% of surveyed participants found the experience positive and reported ease of operation
[71]	11	Untrained (Teleguidance vs. No Assistance)	Image Quality, Learning Experience	All images were deemed adequate for clinical decision-making | Teleguidance through social media app improved diagnostic accuracy and practitioner confidence

**Table 4:** Performance of commercially available medical CMUT devices in cardiovascular US imaging applications.

Cite	n	Comparator	Outcome Measure	Performance
[81]	6	Pre- and Post-training Comparison	Learning Experience	Skill test score improvement: pre-training to 8-week post-training: +2.11 points (95% CI: 1.22–3.00, ES: 1.13); knowledge test improvement: +19.6 points (95% CI: 15.4–23.8, ES: 2.24)
[29]	1	LA435 PZT Probe	Image Quality	No conclusive result
[82]	54	Pre-training vs. Post-training	Learning Experience	Improvement between pre- and 8-week post-training: mean difference = 2.11 (95% CI, 1.22–3.00); ICC for interrater reliability = 0.93 (95% CI, 0.76–0.97)
[80]	75	Vivid q™ and O’Dive™	Diagnostic Performance, Correlation	Moderate agreement produced a smaller number of quality images and less Se and Sp than Vivid q but higher than O’Drive. Overall, it is not a replacement for Venous gas Emboli assessment
[73]	23	Physical Examination	Diagnostic Performance	Se = 91% (95% CI: 89–93%), Sp = 90% (95% CI: 86–93%), PPV = 97% (95% CI: 95–98%), NPV = 77% (95% CI: 72–81%), and DA = 0.94 (95% CI: 0.91–0.96, *p* < 0.001)
[74]	72	Right heart catheterization	Diagnostic Performance	Se = 70.6%; Sp = 85.5%; NPV = 90.4%
[76]	41	Right heart catheterization	Diagnostic Performance	DA: (non-obese = 0.923 and obese = 0.852)
[83]	159	-	-	No conclusive result
[77]	76	Right Heart Catheterization (RHC) vs. Point-of-Care Ultrasound (POCUS) uJVP	Correlation	HR for uJVP ≥10 cm = 3.21 (95% CI: 1.05–9.82, *p* = 0.041); HR for Right Atrial Pressure (RAP) ≥ 10 mmHg = 3.22 (95% CI: 1.05–9.86, *p* = 0.04). Neither uJVP nor RAP was predictive of 90 or 180-day HF hospitalizations
[78]	42	-	Diagnostic Performance	Ultrasound-guided AVF cannulation was feasible and showed a reduction in infiltration rates from 14% to 10.2%, with further protocol improvements lowering rates to 1.7%
[79]	59	High-End US System: Mindray TE7	Diagnostic Performance	First-pass cannulation success rate: Mindray TE7 = 92.59% vs. standard US = 68.75% (*p* = 0.023), indicating significantly better first-attempt success using Mindray TE7
[72]	1	-	-	No conclusive result
[75]	1	-	-	Guided arterial injection
[84]	44	Clinical hypovolemia techniques	Correlation	No significant correlation between clinical hypovolemia and Adjunct Diagnostic Techniques (κ = −0.045), US-IVC (κ = −0.009), or US-C (κ = 0.029)
[85]	26	Experienced Physical Examination	Correlation, Learning Experience	Good correlation (r = 0.73) with an average error of 0.06; ICC = 0.83 (95% CI: 0.44–0.96); moderate-to-high confidence novice satisfaction
[86]	20	-	Diagnostic Performance	12/17 patients had successful pacemaker transplants without major complications. No conclusive result on overall accuracy
[87]	20	Telemed vs. Lumify vs. high-end US (Terason 3300)	Correlation	ICC for inter-observer variability: Telemed = 0.901, Lumify = 0.827, and Butterfly iQ = 0.684
[88]	2	Non-intervention	Diagnostic Performance	BiQ+ was feasible for guiding pericardiocentesis in transit. No conclusive performance statistics provided
[89]	-	ML + BiQ+ Algorithm vs. Conventional Processing	Image Quality	Real-time processing speeds: 60–80 FPS on 288 × 240 input size and 25 FPS on 464 × 208 × 2. Minimal image degradation: mean weight error 0.0054%
[90]	10	Teleguidance vs. No Assistance (Untrained Operators)	Image Quality	Teleguidance group: 100% correctly acquired images with significantly improved quality over time: 76–80% at 2–6 weeks and 53% at 3–4 months follow-up. Time per view: 1–1.5 min, comparable between groups

**Table 5:** Performance of commercially available medical CMUT devices in abdominal and pelvic US imaging applications.

Cite	n	Comparator	Outcome Measure	Performance
[24]	-	PZT Probe	Correlation	Adequate correlation with cellular features, but no conclusive quantitative result
[95]	19	Clinical Diagnosis	Diagnostic Performance	US findings confirmed diagnosis of 50% of the patients and determined new diagnoses in the other half
[93]	114	EPIQ 7 (Philips, US)	Correlation	Nearly perfect correlation (ICC > 0.8) for intra- and inter-operator reproducibility of aortic measurements, except for inter-operator reproducibility at the proximal location (ICC = 0.467)
[97]	30	Pulse Oximetry (PO)	Correlation	Pearson correlation coefficient r = 0.75, *p* < 0.000197
[91]	120	Nonexperts vs. Experts	Diagnostic Performance	Se = 85.7% (95% CI: 42.1–99.6); Sp = 95.5% (95% CI: 88.9–98.8)
[94]	194	-	Image Quality	88.2% excellent/adequate image quality; 96.4% diagnostically interpretable images; 3 h training
[96]	78	Clarius C3	Correlation	ICC = 0.78 (95% CI: 0.62–0.88, p = 0.044) vs. ICC = 0.71 (95% CI: 0.51–0.83, p = 0.011)
[99]	-	Before vs. After Training	Diagnostic Performance	Bladder evaluation and characterization: 25% vs. 53% | Hydronephrosis evaluation: 40% vs. 90% | Diagnosis of pelvic structures: 100%
[92]	17	Clinical Diagnosis	Diagnostic Performance	Confirmed 70% of findings and determined 30% new findings | Challenging to learn
[101]	50	Mindray M9 with a curvilinear 2–5 MHz probe	Diagnostic Performance	Se = 92% (95% CI: 73–99%), Sp = 100% (95% CI: 87–100%), and Diagnostic Accuracy = 96% (95% CI: 85–100%)
[102]	4695	Expert Standard fetal biometry	Learning experience	Novices successfully measured symphysial-fundal height. AI estimator gestational age estimator: Mean Squared Error (MSE) difference = −0.8 days (95% CI: −1.1 to −0.5)
[103]	1	-	-	No conclusive result
[104]	818	High-specification US machine (HSUM)	Correlation	ICC ≥ 0.989. Mean gestational age estimation error: −0.20 days (95% CI, −0.60 to 0.20) in the first trimester (1T); −0.68 days (95% CI, −0.93 to −0.44) in the second/third trimesters (2/3T). Slightly higher mean differences observed with an alternative handheld device but within acceptable limits
[105]	50	Standard bladder scanner (Verathon BVI 9400 and Verathon BladderScan Prime systems)	Correlation	High ICC (0.95 to 0.98) for triplicate measurements using both the standard bladder scanner and the study device. Patient preference: 84% preferred self-measurement
[106]	70	Conventional transvaginal US (GE Logiq P9, Toshiba Xario SSA-660A)	Diagnostic Performance	Se: 92.9% (95% CI: 66.1–99.8) for detecting mispositioned IUDs. Sp: 96.4% (95% CI: 87.7–99.6). PPV: 86.7%. NPV: 98.2%. DA: 95.7%. k = 0.87
[107]	-	-	Learning Experience	Mean pre-test scores improved from 52.8% to 90.6% post-test
[108]	16	CUD	Diagnostic Performance, Correlation	Right hepatic lobe enlargement: Se = 95%, Sp = 87% with CUD| Gallbladder wall thickening: Se = 100% and Sp = 98% | Strong correlation with CUD for measuring the right hepatic lobe (r = 0.912), left hepatic lobe (r = 0.843), portal vein diameter (r = 0.724), and spleen size (r = 0.983) | Substantial agreement in detecting ascites (*p* < 0.0001), gallbladder wall thickening (*p* < 0.0009), and portal vein flow direction (*p* < 0.0001)
[109]	-	-	Learning Experience	All 14 participants scored 100% on clinical exam
[110]	48	High-end US (Canon Aplio i900) vs. Apinion Minisono | Claruis C3 HD3 | iSiniQ 30A | Kosmos | mSonics MU 1 | Philips Lumify | SonoSite iViz | Sonostar Uprobe-C4PL | Vscan Air | Youkey Q7 | BiQ+	Image Quality, Correlation	Among top performers, avg. score 3.83/5 (Acceptable or better in 96%) | High reproducibility and clinical significance score = 3.97/5

**Table 6:** Performance of commercially available medical CMUT devices in neuromusculoskeletal US imaging applications.

Cite	n	Comparator	Outcome Measure	Performance
[124]	1	-	-	No conclusive result
[116]	8	Arthroscopy	Correlation	US detected increased translation but failed to distinguish injury stages
[117]	10	Fluoroscopy	Correlation	US showed moderate correlation with fluoroscopy, but only US could measure the change in distal tibiofibular clear space (TFCS)
[118]	8	Fluoroscopy	Correlation	TFCS distance correlated. US was more sensitive in evaluation
[115]	1	Ophthalmologic examination	Diagnostic Performance	Scans confirmed examination findings
[125]	-	L12-3v and L25e	Image Quality	L12-3v: better in lateral and axial resolution; deeper penetration. L25e: better in detail and contrast resolution
[126]	1	-	-	Best images acquired using pediatric abdomen mode
[121]	-	Musculoskeletal	Learning Experience	No conclusive result
[113]	32	Samsung HS40	Correlation	97% agreement between BiQ and CUD in B-mode imaging. κ = 0.90 (95% CI: 0.89–0.94). No Power Doppler (PD) signal detected by BiQ
[127]	1	-	Learning Experience	Teleguidance was successful for patient care by a US-inexperienced physician; good clinical images and outcomes were obtained
[120]	8	GE Logiq E (CUD)	Correlation	Pearson product-moment correlation = 0.76 (*p* < 0.001)
[112]	66	L64	Diagnostic Performance, Image Quality	Similar diagnostic performance; higher image panoramicity and deep structure definition; worse superficial structure evaluation and Doppler signal
[128]	1	-	Diagnostic Performance	Detected a cortical bulge on the dorsal aspect of the distal right radius using a Butterfly iQ device connected to an iPhone. Confirmed as a torus fracture on radiographs
[129]	20	-	Diagnostic Performance	Saline successfully injected into the Intermediate Temporal Fat Pad (ITFP) 90% of the time using ultrasound guidance. Study focused on anatomical and clinical localization of ITFP for filler injections
[130]	-	-	Diagnostic Performance	No conclusive result
[119]	7	-	Diagnostic Performance	Surgery was successful in all patients
[122]	-	Expert US Instructors (UI) vs. Student Tutors (ST)	Diagnostic Performance	Identification accuracy = 89.2%; no significant difference between both groups
[131]	1	-	-	-
[132]	132	Teleguidance vs. Unguided	Learning Experience, Diagnostic Performance	Training 2-6w: 80% adequately acquired images | Landmark identification: 55% | Perfect image quality | Teleguidance did not affect speed
[133]	-	-	-	-
[114]	1	Conventional PZT Ophthalmic Probe	Diagnostic Performance	Similar diagnostic performance
[123]	1	-	Learning Experience	No conclusive results
[134]	139	US guidance vs. landmark technique	Diagnostic Performance	3.1 min (3.2) vs. 6.3 (7.5); *p* = 0.009 | 1.0 (0.6) vs. 2 (1.3); *p* = 0.29 | 1.0 (0.8) vs. 3.0 (1.3); *p* < 0.001 | −0.15 (0.37) 95% confidence interval [−0.07, −0.23]
[25]	1	-	Image Quality	Vein clearly distinguished
[26]	1	-	Image Quality	Clear 3D view of the leg vein, containing high anatomical information
[135]	10	-	Diagnostic Performance	Dynamic scanning with BiQ identified complete medial patellofemoral complex injury: Se = 77.8%, Sp = 100%, and Acc = 88.9%
[136]	8	-	Diagnostic Performance	Diagnostic Accuracy = 0.97; Se = 100%; Sp = 94.1%; dynamic BiQ quantified medial knee injury severity
[137]	80	-	-	No conclusive result
[138]	7	-	Image Quality	Identified shunt location and measured skin thickness accurately
[139]	33	Hitachi HI VISION Avius	Correlation	Intra-operator reproducibility was almost perfect for both operators on both machines (ICC > 0.80) | Inter-system reproducibility ICC ranged from 0.815 to 0.927 | Measurement difference: 1.8% to 6.6%. Mean muscle thickness difference: clinically acceptable
[140]	7	EyeCubed v3 | Accutome B-Scan Pro	Diagnostic Performance, Image Quality	Both imaged features as small as 0.1 mm with comparable resolution | Similar imaging quality | 1/3 retina specialists showed a statistically significant preference for COU regarding resolution, detail, and diagnostic confidence
[141]	42	SonoSite M-Turbo	Correlation	Excellent inter-device reliability: ICC = 0.92 (95% CI: 0.87–0.94) | ICC (BiQ): ICC = 0.85 (95% CI: 0.73–0.92) | ICC (SonoSite): ICC = 0.89 (95% CI: 0.82–0.93)
[142]	10	CUD	Correlation, Diagnostic Performance	Successfully constructed 3D images for all 10 peripheral nerve blocks | 3D imaging provided enhanced visualization of local anesthetic spread, improving needle direction and placement of anesthetics | Effective postoperative analgesia was achieved in all patients without complications | Time per scan: <5 s.
[143]	19	CT	Diagnostic Performance	Successfully detected the presence of a radial artery pseudo-aneurysm | Effective monitoring of HHUS, allowing for timely intervention | Findings consistent with CT
[144]	82	US guidance vs. Landmark Technique	Diagnostic Performance	3.1 min (3.2) vs. 6.3 min (7.5); *p* = 0.009 (shorter procedure time) Insertion attempts: 1.0 (0.6) vs. 2 (1.3); *p* = 0.29 Needle redirections: 1.0 (0.8) vs. 3.0 (1.3); *p* < 0.001 Depth: −0.15 (0.37) 95% CI [−0.07, −0.23]
[145]	3	Clarius L15 | Clarius L20 | Lumify | Vscan Air	Image Quality	L20 > L15 > Vscan Air > BiQ+ > Lumify. L20 had the best overall image quality for retina, orbicularis oculi muscle, and lacrimal gland imaging. L20 was also the best in vascular imaging. Longer battery life and better stability noted for Lumify and BiQ+

**Table 7:** Performance of commercially available medical CMUT devices in general imaging US applications.

Cite	n	Comparator	Outcome Measure	Performance
[147]	50	Phillips Sparq	Image Quality, Diagnostic Performance	No significant difference in RUSH exam time (249.4 s vs. 251.4 s, *p* = 0.81); similar image quality (82% vs. 86%, *p* = 0.786); κ = 0.69.
[150]	-	Lumify and HHUS	Image Quality, Learning Experience	Selected for cost and single-probe ability to visualize deep and superficial structures but preferred Lumify for imaging heart, lung, and abdomen; novice US medical students could accurately identify anatomical structures with good image quality after 6 h workshops.
[151]	-	-	Learning Experience	Post-test scores significantly improved (*p* = 0.0002 for content; *p* = 0.0001 for ultrasound proficiency); no conclusive result for long-term knowledge retention.
[21]	1	-	-	No conclusive results.
[100]	-	Traditional Ultrasound Machines	Image Quality, Satisfaction	Comparable image quality to traditional ultrasound devices; real-time AI guidance improved ease of use; no conclusive result for full clinical diagnostic accuracy.
[152]	-	Physical Examination	Diagnostic Performance	Ultrasound-guided de-roofing identified larger disease areas than clinical examination; reduced recurrence rates (14% vs. 30%); no conclusive result for direct diagnostic performance.
[6]	29	GE LOGIQ S7 Expert with: SP-D, C1-5-D, and ML5-15 transducers	Image Quality, Diagnostic Performance	UHP was performed faster by 2 min with similar diagnostic image quality.
[20]	33	-	Image Quality	Good image quality for most applications but poorer cardiac imaging due to phased-array limitations. No conclusive result for cardiac function accuracy.
[98]	1000	Standard Diagnostic Methods	Diagnostic Performance	OCUS was useful in early detection of pneumonia and pyelonephritis but required additional confirmation through CT/MRI; Se and Sp not consistently quantified; no conclusive result for direct diagnostic performance.
[32]	110	Clinical Diagnosis	Image Quality, Diagnostic Performance	IVC collapsibility index (CI) ≥ 50% correlated with hypovolemia signs; lung ultrasound B-patterns associated with pulmonary oedema; no conclusive result for general diagnostic accuracy.
[149]	3	Vscan Air| Lumify|Kosmos	Satisfaction, Image Quality	Lumify had the best image quality; Vscan Air rated highest for ease of use; no single handheld device was superior in all categories.
[153]	1	Expert vs. non-expert	Learning Experience, Satisfaction	Veterinary students successfully obtained diagnostic-quality images; Color Doppler and M-mode were used; no conclusive result for comparative performance or long-term retention.
[154]	16	-	Diagnostic Performance	Sonographic lesions were identified in 10 patients (62.5%). Subclinical lesions were identified in 2 patients (12.5%).
[155]	-	Traditional PZT transducers	Image Quality	CMUT-based imaging enhanced 3D volume scanning with higher resolution and contrast; improved penetration depth compared to PZT; no conclusive result for diagnostic accuracy in clinical applications.
[156]	-	In-person vs. Virtually guided	Image Quality, Diagnostic Performance	100% of participants successfully completed the FAST exam; Tele-ultrasound group took significantly longer (341 s vs. 62 s, *p* < 0.001); no conclusive result for long-term retention or diagnostic accuracy improvement.
[157]	-	-	Satisfaction	Convenient cloud storage, personalized cloud folders and easy image access, and prompt feedback from instructors | L: Apple-only compatibility.
[158]	60	Micro Focus X-ray Imaging	Diagnostic Performance	Identification Accuracy: 62.3% vs. 97.6%.
[159]	-	-	Learning Experience	Improved anatomy understanding in 90% of the participants.
[27]	-	Siemens PZT Probes	Image Quality	CMUT integration improves contrast resolution and 3D imaging, but no quantified diagnostic accuracy was reported (no conclusive result).
[148]	74	Sonosite M-Turbo	Image Quality	No differences in resolution or quality but better detail. Mean differences: (Resolution) 1.7, (Detail) 1.6, and (Quality) 1.1).
[160]	-	-	Learning Experience, Satisfaction	100% completion rate; 98% found POCUS training important for emergency medicine; most useful activities: bedside ultrasound and image review; no conclusive result for diagnostic accuracy improvement.
[161]	18	Sonosite M-Turbo	-	-
[162]	2	-	Diagnostic Performance, Satisfaction	Trainees successfully obtained ultrasound images of 12 regional block sites; virtual guidance rated highly for hands-on skill improvement; no conclusive result for superiority over traditional workshops.
[163]	604	Hitachi Arietta V70	Learning Experience, Correlation	Equivalent education tool as a standard US machine. No statistically significant differences in any measurements, including number of correctly acquired and clinically useful images, except for faster EPSS vascular setup time and lower image quality median score.
[164]	174	-	Diagnostic Performance	TCD patterns correlated with patient outcomes; Hyperemia and Posterior High Flow had best prognosis (PCPC 1–2 in 77–89%); Low Flow had worst outcomes (PCPC 1–2 in 42%, *p* < 0.001); no conclusive result for clinical treatment impact.
[165]	29	-	Satisfaction	61.9% of educators were satisfied with image acquisition and interpretation.
[166]	40	-	Learning Experience, Satisfaction	Significant improvement in confidence and knowledge after workshops; increased likelihood of POCUS use in cardiac section; no conclusive result for long-term retention or clinical impact.
[167]	169	Prehospital vs. Hospital	Diagnostic Performance	Prehospital diagnosis confirmed in 66 cases (39.1%); overall diagnostic accuracy = 75.8%; no conclusive result on impact of prehospital POCUS on patient outcomes.
[28]	-	PZT Probe	Image Quality	Better axial resolution than PZT probes; inferior Doppler imaging performance; no quantitative diagnostic accuracy provided (no conclusive result).
[168]	5	Expert Review	Learning Experience	POCUS confidence improved over time; trainees found POCUS useful for early treatment decisions; no conclusive result on direct impact of training on patient care.
[169]	366	-	Satisfaction, Learning Experience	95.6% of scans assisted in decision-making; 65.8% changed patient management (most common change: medication adjustment); no conclusive result on direct patient outcome improvement.
[170]	23	Senior Residents vs. Interns	Learning Experience	91% of residents were highly interested in POCUS; confidence improved in all POCUS skills (*p* < 0.01) except pulmonary embolism (*p* = 0.084); image interpretation skills improved only for pneumothorax detection (*p* < 0.05); no conclusive result on image acquisition improvement.
[171]	4150	Expansion Vibration Lipofilling	Diagnostic Performance	Confirmed subcutaneous-only fat graft placement | Operative times were 6 min shorter | US guidance safter | Lower complications.
[172]	110	-	Learning Experience	Students scored an average of 1.79/2 on image acquisition; 78% average on quiz; 72% reported improved clinical reasoning, 69% improved pathophysiology understanding, and 55% improved patient care; no conclusive result on impact on real patient care.
[173]	438	Untrained vs. Senior Physicians	Learning Experience	4 h of training is sufficient for substantial agreement in sonographic findings in diagnosing heart failure (85%) and moderate for diagnosing tuberculosis (78%) | Se and Sp for pleural effusion: 88% and 95% | Se and Sp for heart failure: 82% and 84%.
[174]	381	Radiology imaging (CT, MRI, US)	Diagnostic Performance	Overall sensitivity: 86.4% (95% CI: 77.0–92.5), specificity: 82.3% (95% CI: 74.1–88.3); cardiac image quality lower than lung (*p* = 0.002); no conclusive result on impact in emergency care.
[175]	40	8 HHUS devices vs. Canon Aplio i900	Image Quality, Correlation	GE Vscan Air achieved highest scores in B-scan quality (3.90 ± 0.65) and clinical significance (4.03 ± 0.73) | Significant variability in B-scan quality among the eight HHUS devices tested | All HHUS devices performed acceptably for certain clinical applications | Some devices showed limitations that could impact their clinical utility.
[176]	-	Teledidactic vs. Hands-on Teaching	Learning Experience	No significant difference in final exam scores (*p* > 0.05); Teledidactic students performed better in FAST (*p* = 0.015) and aorta (*p* = 0.017) modules; no conclusive result on impact in real patient care.
[177]	422	Mono-plane vs. Bi-plane	Diagnostic Performance	1st attempt success rate: 68.3% vs. 73.3% (*p* = 0.395) | Success rate: 100% for both | Median time: 45 s vs. 35 s (*p* = 0.03).
[178]	50	In-person Sonosite vs. Remote Guidance BiQ	Correlation, Learning Experience	No significant difference in assessment scores (*p* = 0.349); no significant difference in assessment duration (482.6 s vs. 432.6 s, *p* = 0.346); no conclusive result on whether virtual training improves real-world ultrasound proficiency.
[179]	-	Dual Stage vs. Traditional Beamforming Techniques	Image Quality	Beamforming was up to 9.23 times faster while maintaining equivalent image quality; no significant degradation in FWHM or FWTM; no conclusive result on clinical diagnostic performance.
[180]	30	Untrained (Teleguidance vs. No assistance)	Diagnostic Performance, Image Quality	Heart (3.46 s vs. 134.1 s), right kidney (55.1 s vs. 149.3 s), and gallbladder (48.5 s vs. 169.3 s) | Higher predicted mean image quality scores for the heart (3.46 vs. 1.86), right kidney (4.49 vs. 1.58), and gallbladder (3.93 vs. 1.5) compared to the control group (*p* < 0.0001 for all).
[181]	60	Micro-focus X-ray imaging	Diagnostic Performance, Correlation	Micro-Focus X-ray identified 97.6% of foreign bodies with 96.5% inter-rater reliability; POCUS identified only 62.3% of foreign bodies with 70.8% inter-rater reliability; no conclusive result on impact beyond detection rates.

**Table 8:** Future work of commercially available CMUT-related modern studies for the years of 2023 and 2024.

Cite	Y	Future Work
[139]	23	Explore the use of HHUS in pathological conditions associated with muscle wasting, such as sarcopenia and cachexia, and validate these findings in a broader range of clinical scenarios and populations, including elderly and critically ill patients. Additionally, further studies could investigate the ergonomics and user-friendliness of HHUS in diverse healthcare settings.
[104]	24	Focus on understanding the underlying causes of the modest underestimation trend and exploring the use of HHUS in a broader range of clinical scenarios and populations. Additionally, studies could investigate the impact of operator training and experience on measurement accuracy, as well as the long-term outcomes of using portable US machines in routine prenatal care.
[171]	23	Focus on refining the technique further and expanding its application to other types of body contouring surgeries. Additionally, long-term studies are needed to evaluate the durability of the aesthetic results and the overall patient satisfaction with this method. Integrating Static Injection, Migration, and Equalization into standard clinical practice could set a new benchmark for safety and outcomes in cosmetic surgery.
[140]	24	Focus on larger clinical studies to further validate the findings and explore the use of portable US devices in various ophthalmic conditions. Additionally, integrating these devices into routine clinical workflows could enhance their utility and impact on patient care. Expanding the evaluation to include a broader range of pathologies and more diverse patient populations will help establish the robustness and generalizability of these findings
[67]	24	Focus on larger, multicenter studies to validate these findings and explore the long-term impact of LUS on patient outcomes, such as reducing hospitalizations and improving quality of life for heart failure patients. Additionally, expanding training programs to include other healthcare providers and settings could further enhance the adoption and utility of LUS in managing heart failure.
[172]	24	Focus on expanding this curriculum to other clinical disciplines and evaluating its long-term impact on students’ clinical practice. Additionally, the use of advanced technologies such as AI and augmented reality for US training could further enhance the learning experience and reduce the need for faculty involvement. Integrating such curricula into medical education programs can help produce more competent and confident physicians, ultimately improving patient care.
[68]	24	Focus on larger-scale studies to further validate these results and explore the use of POCUS in other clinical scenarios. Additionally, developing standardized training programs for pediatricians and other healthcare providers on the use of POCUS will be crucial for its widespread adoption. The potential for POCUS to provide earlier detection of respiratory pathologies and reduce the reliance on chest x-ray makes it a valuable tool in pediatric care.
[105]	24	Focus on longitudinal assessments of postvoid residual bladder volume self-measurement reliability and agreement with provider measurements over time. Additionally, further studies are needed to evaluate the accuracy of patient self-measured postvoid residual bladder volume compared to bladder catheterization volumes, the gold standard for bladder volume measurement. Integrating portable US devices into remote health monitoring systems could enhance patient autonomy and reduce the need for frequent clinical visits, ultimately improving patient outcomes and healthcare efficiency.
[173]	24	Focus on optimizing training protocols to further improve diagnostic accuracy and exploring the use of telemedicine to support remote diagnoses. Additionally, expanding the study to include a broader range of conditions and settings will help validate these findings and enhance the generalizability of the results. Integrating handheld ultrasonographic devices into routine clinical practice could significantly improve healthcare access and outcomes in underserved areas.
[84]	24	Explore the integration of multiple diagnostic modalities to improve the assessment of hypovolemia and fluid responsiveness. Additionally, further studies should investigate the role of acute cardiac dysfunction in the management of hypovolemia and the development of more reliable and comprehensive diagnostic tools. Enhanced training and standardized protocols for the use of US in critical care could also improve the consistency and accuracy of these assessments.
[174]	24	Focus on improving the diagnostic accuracy of HHUS for conditions where its sensitivity is currently lower. Additionally, integrating HHUS into routine ED workflows and training programs can enhance its effectiveness and reliability, ultimately improving patient care and outcomes. Further studies comparing different HHUS platforms and their performance across various clinical settings will help refine best practices and guide the adoption of these technologies in emergency medicine.
[175]	24	Focus on optimizing the use of these devices and exploring their integration with artificial intelligence to enhance diagnostic accuracy. Additionally, further studies comparing HHUS devices in different clinical scenarios and settings will help establish standardized guidelines for their use. As HHUS technology continues to evolve, ensuring consistent quality and reliability across devices will be crucial for their widespread adoption in critical care environments.
[106]	24	Focus on larger-scale studies to validate these results further and explore the use of POCUS in other gynecological and obstetric applications. Additionally, training programs for gynecologists on the use of POCUS could enhance its adoption and improve patient care, especially in low-resource settings where access to conventional US may be limited.
[176]	24	Focus on larger-scale studies to validate these results and explore the long-term retention of skills acquired through teledidactic methods. Additionally, the development of standardized curricula and assessment tools for teledidactic US education will be crucial for ensuring consistent and high-quality training across different institutions. The adoption of portable US devices like the BiQ in teledidactic programs could further enhance the accessibility and effectiveness of US education worldwide.
[69]	23	Focus on further optimizing TUS protocols, training, and technology to address its limitations and enhance its diagnostic accuracy. Expanding the use of TUS in other clinical settings and interventions could also be explored to maximize its benefits across various medical fields.
[141]	23	Explore the reliability of handheld US technology in measuring other musculoskeletal and pathological structures. Additionally, training programs for clinicians on the use of handheld US devices could enhance their adoption and integration into routine practice, ultimately improving patient care and clinical outcomes.
[142]	23	Explore the broader application of this technology in other regional anesthesia procedures and in settings where access to full-sized US equipment is limited. Additionally, further research could focus on refining the 3D imaging capabilities of handheld devices to enhance their utility in complex or anatomically challenging cases.
[177]	23	Explore the impact of operator experience on the effectiveness of these techniques, as well as the potential benefits of bi-plane imaging in other clinical scenarios beyond PIV catheterization.
[178]	24	Explore the long-term retention of skills acquired through virtual instruction and investigate the applicability of this training method in other medical specialties. Additionally, developing standardized assessment tools for evaluating US skills could improve the consistency and reliability of training outcomes.
[179]	23	Explore further optimization of the beamforming algorithm, as well as its application to other types of US transducers and imaging scenarios. Additionally, expanding the method’s use in clinical settings could validate its utility and performance in a broader range of medical applications.
[143]	23	Explore the broader use of handheld US devices in diagnosing other vascular conditions and their role in outpatient follow-up care. Additionally, research into the integration of these devices into routine clinical workflows could enhance their adoption in various medical settings, particularly in resource-limited environments.
[107]	23	Explore the long-term impact of such training on patient outcomes, as well as the integration of POCUS into routine obstetric care. Additionally, similar training models could be adapted for other clinical applications of POCUS, further expanding the reach of portable US technology in resource-limited settings.
[85]	23	Explore the long-term retention of these skills and assess the impact of US-based JVP measurement on clinical outcomes. Expanding this training to include a wider range of diagnostic applications for point-of-care US (POCUS) could further enhance the diagnostic capabilities of novice clinicians.
[86]	23	Explore long-term outcomes of Cardioneuroablation and the role of advanced imaging techniques, such as those provided by portable US devices, in guiding these procedures.
[87]	23	Focus on improving the inter-observer reliability of devices like the BiQ through enhanced training programs or technological improvements. Additionally, expanding the use of these devices to other vascular measurements and exploring their use in patients with known cardiovascular conditions could further validate their utility in clinical practice.
[70]	24	Focus on expanding this model to include more diverse patient populations, different pathologies, and other types of US exams. Additionally, further exploration into improving the inter-rater reliability and the effectiveness of remote training methods could enhance the overall utility of self-administered US in home-based care.
[108]	23	Focus on larger-scale studies to further validate these findings and explore the use of HHUS in other transplant-related complications or in different clinical settings. Additionally, integrating HHUS with telemedicine could enhance its utility in remote or underserved areas, providing critical diagnostic capabilities where conventional US is not available.
[180]	24	Explore the application of this technology in different clinical scenarios, including emergency medicine and home healthcare. Additionally, further studies could assess the long-term effectiveness of teleguidance in various populations and explore ways to optimize the technology for broader clinical adoption.
[144]	24	Explore the long-term outcomes of infants diagnosed with intracranial hemorrhage using cranial POCUS and evaluate the impact of this early detection on neurodevelopmental outcomes. Additionally, expanding the training program to include more neonatal intensive care units and different types of POCUS applications could further validate the utility of handheld US devices in neonatal care.
[109]	23	Explore the long-term impact of such training on patient care and outcomes, as well as the potential for expanding the program to include other medical specialties. Additionally, further studies could investigate the integration of telemedicine with POCUS training to provide continuous support and guidance for healthcare providers in remote areas.
[52]	23	Focus on scaling this approach in different geographic regions, evaluating its long-term impact on breast cancer outcomes, and exploring its applicability to other types of US imaging. Additionally, further studies could investigate the cost-effectiveness of widespread Volume Sweep Imaging deployment compared to traditional breast cancer screening methods in middle-income countries.
[145]	24	Explore the clinical implications of using these devices in actual patient settings, particularly in ophthalmology and facial aesthetics, and assess their long-term reliability and user satisfaction. Additionally, further studies could evaluate the impact of device selection on clinical outcomes, particularly in procedures where imaging precision is critical.
[181]	23	Focus on optimizing micro-focus X-ray imaging technology for broader clinical use, evaluating its effectiveness in other types of foreign body detection, and exploring its integration with artificial intelligence to further enhance diagnostic accuracy. Additionally, studies involving human subjects could validate these findings and assess the practical feasibility of implementing micro-focus X-ray imaging in various clinical environments.
[88]	24	Focus on developing guidelines for the use of US-guided pericardiocentesis in transport, particularly in patients with aortic dissection, and exploring the use of advanced imaging techniques to better assess the risks of intervention in these cases. Additionally, further studies could investigate the outcomes of patients who undergo pericardiocentesis during transport to identify factors that may improve survival rates in such critical conditions.
[89]	24	Explore the application of this technology in more complex imaging scenarios, such as harmonic imaging and 3D US. Additionally, further optimization of deep learning models for different clinical applications could enhance the versatility and effectiveness of handheld US devices in various medical fields.
[90]	23	Focus on expanding the study to a larger cohort, exploring the long-term outcomes of such an approach, and developing standardized protocols for patient training and teleguidance in various clinical settings.
[71]	23	Focus on scaling this approach, evaluating its long-term impact on patient outcomes, and exploring ways to integrate it into routine healthcare practices in low- and middle-income countries. Additionally, studies could investigate the effectiveness of different training models and feedback mechanisms to optimize the learning curve for local providers.
[110]	23	Focus on expanding the range of clinical applications, exploring the use of these devices in more specialized sonographic questions, and further comparing their performance with other emerging handheld technologies. Additionally, studies could investigate the long-term impact of using handheld devices on diagnostic accuracy and patient outcomes in various clinical settings.

## Supplementary Materials

The following supporting information can be downloaded at https://www.mdpi.com/article/10.3390/s25072245/s1, an Excel file including additional data extracted in the review has been attached to this submission.
